# Trichomoniasis Infection and Associated Risk Factors Among Women of Reproductive Age in Nepalgunj Medical College Teaching Hospital, Kohalpur, Banke

**DOI:** 10.1155/japr/7844554

**Published:** 2025-09-27

**Authors:** Yamini Chhetri, Rajendra Pd. Parajuli, Mahendra Maharjan

**Affiliations:** ^1^Central Department of Zoology, Institute of Science and Technology, Tribhuvan University, Kirtipur, Kathmandu, Nepal; ^2^Herbert Wertheim School of Public Health and Human Longevity Science, University of California San Diego (UCSD), San Diego, California, USA

## Abstract

**Background:** *Trichomonas vaginalis* is a flagellated, protozoan parasite causing a curable sexually transmitted disease, trichomoniasis. The prevalence of the infection has been increasing globally and nationally, although epidemiological studies are scarce in Nepal.

**Objective:** This study is aimed at evaluating the prevalence of trichomoniasis and its associated risk factors among women of reproductive age visiting the gynecological outpatient department of Nepalgunj Medical College Teaching Hospital, Kohalpur, Banke.

**Methods:** The study was conducted among 290 women of reproductive age attending the hospital's Gynecology Outpatient Department (GOPD). Structured questionnaires were employed for data collection on demographic, clinical characteristics, and associated risk factors. Participants were included using consecutive sampling techniques. A vaginal swab sample was collected using a sterile cotton swab and tested using a direct wet mount. Data analysis was performed using R Core Team (2023), employing bivariate and multivariate approaches. Statistical significance was determined by *p* value < 0.05, with a 95% confidence interval (CI).

**Result:** The overall prevalence of trichomoniasis was 13.79% (95% CI, 9.52–18.07). The higher prevalence was found among women aged 36-45 years (17.46%), with school-level education (16.50%), married (14.23%), municipality residents (14.69%), and working women (24.56%). In the multivariate analysis, women engaged in occupations outside the home (e.g., laborers) had significantly higher odds of trichomoniasis compared to housewives or students (adjusted odd ratio (aOR): 9.36; 95% CI: 2.82–31.06). No other sociodemographic or behavioral characteristics remained significantly associated after adjustment. Clinical symptoms independently associated with the infection included elevated body temperature (> 98.6°F) (aOR: 15.89; 95% CI: 5.07–49.76), greenish vaginal discharge (aOR: 7.49; 95% CI: 2.73–20.54), and foul-smelling discharge (aOR: 10.18; 95% CI: 3.34–31.03).

**Conclusion:** This study revealed a higher prevalence of *T. vaginalis* among women of reproductive age, specifically those who engaged in occupations outside the home (e.g., laborers). This could lead to other complications; hence, routine screening regardless of symptoms and awareness campaigns to the general public are advised to minimize the risk of infection.

## 1. Introduction

Trichomoniasis is the most common, nonviral sexually transmitted infection (STI) caused by an aerobic, flagellated, protozoan parasite, that is, *Trichomonas vaginalis* [1]. It is a parasite infecting the human urogenital tract, causing different complications. Humans are the only reported hosts of the infection [2]. Unlike many protozoan parasites, *T. vaginalis* possesses a trophozoite form and lacks a cyst stage in the life cycle [3]. Rare instances of nonsexual transmission exist, while the primary mode of transmission is via sexual contact, including vaginal, anal, or oral sex [4, 5]. Despite trichomoniasis being assumed to be the most common treatable STD in the world, it is difficult to quantify trichomoniasis incidence due to the high incidence of asymptomatic infection. Symptomatic women with trichomoniasis usually complain of typical green, frothy, malodorous vaginal discharge, vaginal irritation, lower abdominal pain, vulvovaginal secretion or soreness, dysuria, dyspareunia, discomfort, pelvic inflammatory disease (PID), cervical neoplasia, and infertility [4, 6]. The adhesion of *T. vaginalis* to vaginal epithelial cells plays a role in increasing the chance of infection and transmission of the human immune deficiency virus (HIV) [3, 7]. In pregnant women, the infection causes abortion, preterm rupture of membranes, preterm delivery, and delivery of low-birth-weight infants [7, 8].

Trichomoniasis is the most common STI with an estimated overall annual incidence of 156 million cases globally among people aged 15–49 years old. Among females, the infection is estimated at 73.7 million cases, with the majority asymptomatic and 50% experiencing frothy and foul-smelling vaginal discharge [9]. The disease has easy diagnosis and prevention; however, the prevalence remains high globally and nationally. The WHO stated in 2020 that the global burden of trichomoniasis in women was reported from the African region (12%), followed by America's region (7.1%), Eastern Mediterranean region (4.7%), Western Pacific region (3.7%), Southeast Asia region (2.7%), and European region (1.7%). The prevalence among females (4.9%) was higher than males (0.5%) [10].

The comprehensive data on the prevalence of *T. vaginalis* infection in Nepal remains scarce, due to the lack of screening programs and healthcare resources. HIV poses a formidable challenge in Nepal, while preventive measures specifically targeting trichomoniasis have yet to be implemented [11]. Hence, trichomoniasis can be considered a neglected disease [12].

The increased rate of trichomoniasis is attributed to various factors such as poor personal hygiene, multiple sexual partners, and low socioeconomic level [13]. However, untreated trichomoniasis can cause adverse birth outcomes and uncommon perinatal transmission leading to vaginal and respiratory infections in newborns [14]. The trichomoniasis infection has increased, especially in developing countries and people who are engaged in high-risk behaviors such as poor sexual activity, unprotected sexual activities, and having more than one sexual partner. The failure and resistance of parasites to metronidazole are another risk in eradicating the disease [15].

The cooccurrence of various other STIs such as *Chlamydia trachomatis*, *Neisseria gonorrhoeae*, human papillomavirus (HPV), and also Herpes Simplex Virus Type 2 (HSV-2) [14]. Despite the high global and national incidence, the infection has not gotten the related authorities' attention and public concern. Though it is one of the curable STIs, the risk of contracting other hazardous infections remains persistent. The research based on the association of infection and risk factors is limited in countries like Nepal, as per our knowledge. Most research conducted is concentrated among pregnant women. Therefore, this study's objective is to assess the prevalence of *T. vaginal* and its associated risk factors among women of reproductive age visiting the gynecological outpatient department (GOPD) of Nepalgunj Medical College Teaching Hospital (NGMCTH), Kohalpur, Banke.

## 2. Methodology

### 2.1. Study Area, Design, and Period

The study was conducted at NGMCTH, Kohalpur, Banke. Kohalpur Municipality is located in the Banke District of Lumbini Province, Federal Democratic Republic of Nepal. The municipality consists of 15 wards. It has a city hospital, a city health promotion center, 13 health centers, one Ayurveda dispensary, one sickle cell anemia lab, a private medical college, and three private hospitals [16]. NGMCTH was established in 1996 A.D. in Kohalpur, affiliated with Kathmandu University [17]. It is one of the largest hospitals serving people from different regions of western Nepal. The GOPD of NGMCTH serves an average of 20–40 women per day. The study period was from 18 August 2023 to 12 February 2024.

### 2.2. Study Population and Selection Criteria

The women of reproductive age visiting the GOPD of the hospital with symptoms similar to trichomoniasis, that is, the presence of discharge, dyspareunia, dysuria, lower abdominal pain, malodorous discharge, and so forth, were approached for the research.

### 2.3. Sample Size Determination

The sample size for the study was determined by using Cochran's equation [18]:

 $$\begin{array}{c} n=\frac{z^{2}p\left(1-p\right)}{e^{2}} \end{array}$$

where *n* represents the sample size, *z* is the value corresponding to a 95% level of significance = 1.96, *p* is the proportion of the prevalence of *T. vaginalis* in reproductive women = 0.5, *q* = (1 − *p*) = (1 − 0.5) = 0.5, *d* is the marginal error assumed to be 5.75%, and *n* = (1.96)^2^(0.5)(0.5)/(0.0575)^2^ = 290.48.≈290.

### 2.4. Sampling Technique

This study used a consecutive sampling technique, and two hundred ninety (290) reproductive women attending the GOPD at NGMCTH, Kohalpur during the study period who fulfilled the inclusion criteria were enrolled.

### 2.5. Data and Sample Collection Methods

Informed permission was requested from the participants. An explanation of the research, speculum examination, and questionnaire form was briefed to the participants. The participants signed the written consent form, and the questionnaire form (sociodemographic and clinical characteristics) was filled up by the researcher in face-to-face interviews. Vaginal swab specimens were collected via speculum examination by a trained gynecologist. A sterile high vaginal swab (HVS) was gently inserted through the speculum into the posterior fornix of the vagina and rotated to collect vaginal discharge. To reduce discomfort, participants were asked to void before the procedure. Depending on the caseload, the swab was either directly placed onto a clean slide for immediate microscopic examination or transferred into a sterile collection tube containing normal saline. Since the laboratory was within the same hospital, the samples were generally examined within 30 min of collection. If immediate examination was not feasible, the swabs were stored temporarily at room temperature in sterile saline tubes and promptly transported to the Microbiology Laboratory of NGMCTH for processing.

The questionnaire was initially developed in both English and Nepali languages to ensure consistency.

### 2.6. Laboratory Analysis and Diagnosis

The color of the discharge was observed and noted down. The specimen was then centrifuged for 5 min at 2000–3000 rpm. The supernatant was discarded, leaving a few milliliters of normal saline (0.9% sodium chloride solution) (NS)with the specimen. The specimen was mixed well and placed on a labeled clean, dry glass slide, covered with a coverslip [19]. The wet mount was visualized under the microscope from lower to higher magnification (10x, 40x, and 100x) in search of motile trichomonads [20]. Giemsa helped with the visualization of trichomonads in a subset of initially positive samples. A smear was prepared using the specimen and left to air-dry. The prepared smear was fixed by submersion in absolute methanol for 1 min and then allowed to dry before the Giemsa staining. After being stained with Giemsa dye, prepared fresh using 10% Giemsa working solution from the stock solution (HiMedia Laboratories, India). In a beaker, 9 mL of buffered water (pH 7.2) and 1 mL of the stock were added. Contamination was avoided by taking an aliquot from the large bottle containing the stock solution. The working solution was prepared just before staining the slides and used within a maximum of 15 min of preparation [21]. The stained slide was visualized at 100x magnification using anisole.

### 2.7. Data Processing Analysis

Data Analysis was performed using R Core Team (2023) Version 4.3.2 [22]. Collected data from the questionnaire were encrypted and entered into Microsoft Excel before importing into R for statistical analysis. Missing data were minimal, as all interviews were conducted in person by the lead researcher; when responses were not available, missing values were systematically denoted with a dot for consistency in data entry. Descriptive statistics were used to summarize sociodemographic, clinical, and behavioral characteristics of participants. Results were presented using texts, tables, and graphs. The prevalence of trichomoniasis was calculated using frequency distribution, both in the overall study population and stratified by associated risk factors. Associations between categorical variables were assessed using the Chi-square (*χ*^2^) test or Fisher's exact test, as appropriate.

To identify independent predictors of trichomoniasis, we conducted separate multivariable logistic regression analyses for clinical symptoms and for sociodemographic/behavioral characteristics. For clinical symptoms, only variables that were statistically significant (*p* < 0.05) in the bivariate analysis were included in the multivariable model to estimate adjusted odds ratios (aORs) with 95% confidence intervals (CIs). For sociodemographic and behavioral factors, all candidate variables were retained in the multivariable model, regardless of bivariate significance, to explore their mutually adjusted associations with the outcome. In both models, we assessed multicollinearity using Spearman's correlation, ensuring that no pair of variables had a correlation coefficient (rho) ≥ 0.6. In our study, all women with multiple sexual partners or a strawberry cervix tested positive for trichomoniasis, resulting in infinite odds ratios and unstable CIs; thus, these variables were excluded from the regression analysis. Statistical significance was set at *p* < 0.05.

## 3. Results

### 3.1. Sociodemographic Characteristics

A total of 290 women of reproductive age ranging from 16 to 50 years, with a mean age of 31.13 years and a standard deviation of 7.29 years, were included in this study. Notably, half of the participants (50%) were between the age range of 26 and 35. The prevalence was 13.79% by wet mount and also confirmed by Giemsa staining. The high prevalence was found among women in the 36–45 years (17.46%) age group, followed by the less than 25 age group (16.44%), the 26–35 years age group (11.72%), and no women of more than 46 years age group reported to be positive for trichomoniasis. Despite this trend, no substantial linkage was established between the age category and *T. vaginalis* infection prevalence (*p* = 0.441).

Over 60% of participants achieved secondary education (68.96%), correlating with the increase in infection among school scholars (16.50%). Illiterate women accounted for 8.82% of infections, while college graduates constituted 4.54%. However, initial observation suggested a weak association between academic status and the probability of infection emergence (*p* = 0.157).

Most participants were married (94.48%) and housewives (75.52%). Married women showed a higher infection rate (14.23%), followed by unmarried women (12.5%). Nevertheless, marital status yielded negligible effects on the chance of contracting the infection (*p* = 0.844).

Most participants hailed from urban/municipality areas (72.76%, Table 1). The urban/municipality residents (14.69%) showed higher infection rates than rural municipality resident women (11.40%). Residency and the infection showed no significant association (*p* = 0.568).

Occupation involvement played a decisive factor, though working women showed the highest percentage of infection (24.56%), preceded by scholars (14.29%) and housewives (10.96%). Subsequent probing confirmed a notable association between occupation and *T. vaginalis* infection (*p* = 0.037).

**Figure 1:**
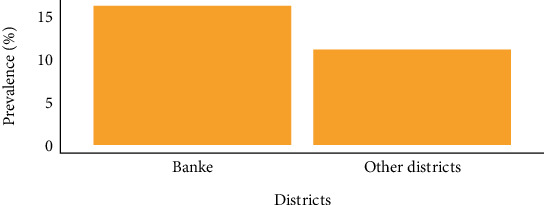
District-wise prevalence of trichomoniasis among women of reproductive age.

At the regional scale, geographic origin exerted little bearing on trichomoniasis infection; nonetheless, the Banke district recorded the largest case count (16%). Comparatively, other district participants (11%) displayed considerably smaller counts (Salyan, Bardiya, Dang, Rukum West, Jajarkot, and Kailali; Figure 1).

Across distinct ethnocultural lineages, both Dalit and Janajati showcased peak infection (17%). In contrast, the lowest prevalence was seen among Brahmins (10%, Table 2).

Regarding economic status and ethnicity, the low-class group of Chhetri/Thakuri (17.39%) and Janajati (22.22%) evidenced considerable infection frequencies. Noteworthily, high-class Brahmins exhibited augmented figures (14.29%), contrasting with predominantly middle-class Dalit infections (85.71%).

### 3.2. Clinical Characteristics

Most participants had normal/lower body temperature (87.93%), out of whom 21 (8.23%) tested positive for trichomoniasis (Table 3). Body temperature and *T. vaginalis* infection strongly correlated (*p* < 0.001). Lower abdominal pain was experienced by 85.52% of participants, with 29 (11.69%) being infected, indicating a significant association (*p* = 0.013). Only 10% of women reported dyspareunia, yet 11 (27.5%) were positive, presenting a remarkable correlation (*p* < 0.001) with the infection. One-third of participants suffered from dysuria (33.79%), though just 18.37% resulted positive, lacking a significant association (*p* = 0.158). Although vaginal itching was widespread (42.41%), merely 24.39% of symptomatic participants acquired *T. vaginalis* infection, showcasing a clear correlation (*p* < 0.001).

Among 74.14% reporting vaginal discharge, 18.61% turned up, reflecting a significant association (*p* < 0.001) to the infection. Various shades of discharge were documented: 25.86% had normal discharge, 7.86% encountered curdy-white discharge, 16.21% with greenish discharge, 41.72% with milky-white discharge, and 8.62% endured mixed discharge. Remarkably, 44.68% of positive women had greenish discharge and 36% of mixed discharge. A strong association was reported between discharge color and trichomoniasis (*p* < 0.001). Women with malodorous discharge (32.41%) were more prone to harbor the parasite (36.17%), denoting a significant relation (*p* < 0.001).

Strawberry cervix was reported by fewer participants (2.41%), and every case coincided with *T. vaginalis* infection. Statistical analysis confirmed a substantial association (*p* < 0.001) between the two factors.

While the vast majority (96.55%) of participants stated they had a single sexual partner, 13.57% of these women still tested positive for the infection. Interestingly, every participant (0.69%) reported having multiple partners who contracted the infection, indicating a meaningful association (*p* = 0.016).

The study population utilized diverse contraceptive approaches: more than half (52.07%) admitted to refraining from using contraception during intercourse. Some utilized hormonal approaches (such as Norplant's and DMPA) accounting for 31.04% of users, while others relied on barrier methods (condoms) representing 9.65%, and few resorted to permanent sterilization measures (3.79%). Nevertheless, no significant correlation was uncovered between the contraceptive method and trichomoniasis susceptibility.

### 3.3. Factors Associated With Trichomoniasis Infection

Clinical symptoms like the presence of higher body temperature (i.e., > 98.6° F) (aOR 15.89, 95% CI [5.07 to 49.76]), report of greenish (aOR 7.49, 95% CI [2.73 to 20.54]), and foul-smelling (aOR 10.18, 95% (CI) [3.34 to 31.03]) discharge were consistently associated with *T. vaginalis* infection during the multivariate adjusted model (Table 4). This study also indicated that *T.* vaginalis-infected participants presented higher odds of vaginal itching (cOR 5.07, 95% CI [2.37 to 10.83]) and dyspareunia itching (cOR 4.90, 95% CI [2.10 to 11.36]) compared to healthy counterparts during the univariate model. Yet, such associations did not persist when adjusted for other significant variables during the multivariate models. Dysuria was not associated (*p* > 0.05) with *T. vaginalis* infection.

The presence of *T. vaginalis* infection was higher among participants who reported worker as their occupation compared to participants reported as other as occupation (i.e., housewife or students) [(aOR 9.36, 95% CI [2.82 to 31.06]) (Table 5). Prevalence of *T. vaginalis* infection was not associated (*p* > 0.05) with any other sociodemographic, lifestyle, or other behavioral characteristics evaluated.

## 4. Discussions

In this study, the overall prevalence of trichomoniasis among the women of reproductive age who visited GOPD was 13.79% (95% CI, 9.52–18.07). The findings were similar to the reports from Nigeria (11%–13%) [23, 24], Maryland (11.8%) [25], Trinidad and Tobago (11.9%) [15], Central India (12.6%) [26], Kosovo (12.56%) [27], Iran (12.9%) [28], and Tanzania (13.3%) [29]. However, some researchers reported a lower prevalence than this study: Uganda (0.28%) [30], Nairobi (0.4%) [31], Egypt (0.5%) [32], Iran (2.4%) [3], Ethiopia (2.56%) [33], Nigeria (5.3%) [34], Island of Bubaque (5.9%) [35], Swaziland (8.4%) [36], India (0.5%–8.1%) [37–40], and even in some reports from Nepal (0.99%–7.10%) [41–44]. The lower prevalence rate could be due to variations in sample size, improved hygienic practices, awareness, geographical conditions, and a high literacy rate. Moreover, this finding was lower than the studies conducted in Iraq (53.03%) [5], Nigeria (42.5%) [13], and South Africa (20%) [45]. While we have compared our prevalence findings with numerous international studies, caution is warranted in directly interpreting these differences. Many of the cited studies vary considerably in terms of population characteristics, healthcare access, diagnostic methods, inclusion criteria, and cultural contexts. Therefore, these comparisons are presented primarily to situate our findings within the broader global and regional landscape rather than for strict epidemiological equivalence.

The maximum prevalence was identified within the age range of 36–45 years (17.46%), which mirrors results published by Saito-Nakano et al. indicating the highest prevalence rate occurring above the age of 30 (39%) [46]. Many of the infected participants in this study were married, consistent with previous research from Agabi et al. in Nigeria [34] and Yadava et al. in India [40].

Our finding that women working outside the home (e.g. laborers or petty traders) had higher trichomoniasis prevalence aligns with several reports from low- and middle-income countries (LMICs). For example, a Tanzanian clinic survey found that ~19.8% of women engaged in petty business (small-scale trading) had *T. vaginalis*, significantly higher than in other occupational groups [29]. Likewise, a recent Libyan study of women attending obstetric clinics showed that 85.1% of those with *T. vaginalis* infection were employed outside the home (vs only 14.9% housewives, *p* = 0.008) [47]. Another study in Kosovo also indicated the highest prevalence of trichomoniasis among the employed (19.71%) and lowest among students (14.81%) and unemployed (7.33%) [27]. These findings suggest that women in labor-intensive or trading occupations often have a higher STI burden, possibly due to greater exposure to partners or settings where condom use is low. Women working outside the home may interact with more social or sexual networks (including transactional sex), or may have partners who also have multiple partners, increasing transmission risk [29, 47]. Moreover, these occupations may correlate with lower socioeconomic status and less access to health education, further raising vulnerability. In contrast, other studies found no such association or even higher *T. vaginalis* rates among housewives. For instance, an Iranian survey of 291 women at health centers found that 91.1% of infected participants were housewives [3]. Similarly, another study in Iran reported no significant relationship between women's jobs and trichomoniasis (*p* = 0.17), and noted that prevalence was slightly higher in housewives [48]. In Ethiopia, a study of pregnant women showed 9.5% infection among employed women versus 7.4% among “unemployed” (predominantly housewives), with a nonsignificant difference (OR≈0.76, *p* = 0.73) [12]. These divergent results could stem from contextual factors. In some settings, housewives may engage in unprotected sex within marriage (and if husbands have outside partners, wives are at risk), while working women may actually have greater STI awareness and condom use [48]. Differences in sampling (clinic-based vs. community surveys), overall low infection rates, and other confounders (age, marital status, and hygiene practices) could also influence whether housewives or laborers show higher prevalence. Overall, while our results agree with other LMIC reports that women working outside the home have higher *T. vaginalis* prevalence, the literature shows mixed findings. The heterogeneity likely reflects differing social patterns, partner behaviors, and sampling methods across settings. These comparisons underscore the need to consider local context when interpreting occupational risk for trichomoniasis and suggest that both working women and housewives may warrant targeted STI screening and education depending on the community.

Among the independent variables, having multiple partners was statistically significant with the infection. Rogers et al. and De Waaij et al. also mentioned multiple partners as a risk factor increasing the chances of *T. vaginalis* infection [25, 45]. Social shifts granting women the ability to make partnership decisions contribute to the increasing number of partners; subsequently increasing the risk of STI infections [35].

In this study, clinical symptoms demonstrated a significant association with the trichomoniasis infection, such as body temperature, dyspareunia, and green-colored malodorous discharge. Consistent with Nateghi et al.'s observation of malodorous discharge [28] and Ghallab et al.'s recordings of discharge presence accompanied by dyspareunia [49]. Furthermore, the presence of such clinical characteristics and no significant association were observed in studies in Iran [28], Egypt [49], Iraq [5], and Nigeria [24]. Yet, these clinical features may be especially valuable for guiding syndromic management protocols in resource-limited settings, where laboratory confirmation is not always feasible.

The method of contraception showed no significant association with trichomoniasis infection in univariate or multivariate model in our study, aligned with the findings of Kohn et al. [3]. This lack of association in our study may reflect inconsistent condom use, as fewer than 10% of participants reported using condoms, and nearly half of them tested positive for *T. vaginalis*. Engaging in unprotected sexual encounters often leads to alterations in the inherent vaginal pH balance, triggering persistent alkalinization and ultimately increasing susceptibility to the infections [30]. Women in informal or low-paid jobs in Asia—often with limited education and resources—face elevated trichomoniasis risk due to poor STI awareness, exploitative work settings, and greater exposure to high-risk sexual networks [50, 51]. These findings underscore the importance of integrating early detection and syndromic management approaches into routine gynecological care, especially in resource-limited settings. Public health strategies should prioritize community-based screening and targeted health education campaigns that address stigma, promote awareness, and encourage timely care-seeking. Particular attention should be given to high-risk groups identified in this study—such as women working outside the home (e.g., laborers or petty traders)—by developing culturally appropriate interventions that improve access to care and reduce the burden of undiagnosed trichomoniasis.

### 4.1. Limitation of the Study

Due to resource limitations, this study enrolled only nonpregnant, reproductive-age women presenting with symptoms at the gynecology outpatient department of NGMCTH. As a result, the findings may not fully represent the broader female population. Moreover, restricting the sample to symptomatic individuals may have introduced selection bias by excluding asymptomatic carriers of *T. vaginalis*, who comprise a significant portion (i.e., > 50%) of cases. This may have led to an overestimation of the true prevalence among women of reproductive age. Moreover, the detection of *T. vaginalis* was based exclusively on direct wet mount microscopy, with Giemsa staining used primarily for visualization and partially for confirmation. These methods are less sensitive than advanced diagnostic techniques such as culture, PCR, or ELISA. Additionally, the cross-sectional nature of the study limits our ability to establish temporal or causal relationships between risk factors and trichomoniasis. Seasonal variation in clinic attendance patterns may also have influenced participant characteristics. Furthermore, several potentially important confounding variables—such as history of other STIs, vaginal douching practices, immune status (e.g., HIV), substance or alcohol use, and menstrual hygiene—were not assessed in this study. The omission of these factors may have introduced unmeasured confounding, potentially affecting the observed associations. In addition, the use of face-to-face interviews to collect sensitive behavioral data—particularly regarding sexual history—may have introduced social desirability bias. Future studies may benefit from using self-administered or anonymous questionnaires to improve the accuracy of responses in this domain. Future research should consider multicenter or community-based studies using more sensitive diagnostic tools and broader inclusion criteria to generate findings that are more representative and generalizable.

## 5. Conclusion and Recommendations

In the present study, the overall prevalence of *T. vaginalis* was 13.79% among women attending the GOPD of NGMCTH, Kohalpur. Elevated odds of trichomoniasis among manual labor women are notable. Awareness campaigns were advised regarding sex education, particularly about safe sexual practices with safe and trusted partners, methods of contraception, and their proper use to prevent STIs (trichomoniasis) to the general public, both men and women. Routine screening for trichomoniasis in symptomatic women should be prioritized in gynecological care. Improving diagnostic capacity with sensitive tools like PCR or culture in regional labs is recommended, along with incorporating STI education into school and community programs to support early detection and prevention.

## Figures and Tables

**Table 1:** Demographic characteristics versus prevalence of trichomoniasis among women of reproductive age visiting gynecological out-patient department, Nepalgunj Medical College Teaching Hospital, Kohalpur, 2024.

**Demographic variables**	**Total no. (%)**	**Positive no. (%)**	**p** ** value**
Age group			
< 25	73 (25.17)	12 (16.44)	0.441^#^
26–35	145 (50)	17 (11.72)	
36–45	63 (21.72)	11 (17.46)	
> 46	9 (3.10)	0 (0)	
Educational status			
Illiterate	68 (23.45)	6 (8.82)	0.157^#^
School level	200 (68.96)	33 (16.50)	
College level	22 (7.59)	1 (4.54)	
Marital status			
Married	274 (94.48)	39 (14.23)	0.844^#^
Unmarried	8 (2.76)	1 (12.5)	
Widow	8 (2.76)	0 (0)	
Residency			
Municipality	211 (72.76)	31 (14.69)	0.568⁣^∗^
Rural municipality	79 (2.24)	9 (11.40)	
Occupation			
Housewife	219 (75.52)	24 (10.96)	**0.037**⁣^∗^
Student	14 (4.83)	2 (14.29)	
Work	57 (19.65)	14 (24.56)	

*Note:* Bold emphasis indicates significance.

⁣^∗^Chi-square test.

^#^Fisher exact test while any cell has a count less than 5.

**Table 2:** Distribution of trichomoniasis among different ethnic groups in relation to economic status.

**Ethnicity**	**Total no.**	**Positive no.**	**Percentage (%)**
Brahmin			
High class	7	1	14.29
Middle class	44	5	11.36
Low class	8	0	—
Total	59	6	10.17
Chhetri/Thakuri			
High class	5	0	—
Middle class	119	16	13.45
Low class	23	4	17.39
Total	147	20	13.60
Janajati			
High class	2	0	—
Middle class	30	5	16.67
Low class	9	2	22.22
Total	41	7	17.07
Dalit (ccheduled caste)			
High class	3	1	33.33
Middle class	7	6	85.71
Low class	31	0	—
Total	41	7	17.07

**Table 3:** Clinical characteristics versus prevalence of trichomoniasis among women of reproductive age visiting gynecological outpatient department, Nepalgunj Medical College Teaching Hospital, Kohalpur, 2024.

**Clinical characteristics**	**Total no. (%)**	**Positive no. (%)**	**p** ** value**
Body temperature			
< 98.6	255 (87.93)	21 (8.23)	**< 0.001**⁣^∗^
> 98.6	35 (12.07)	19 (54.29)	
Lower abdominal pain			
Yes	248 (85.52)	29 (11.69)	**0.013**⁣^∗^
No	42 (14.48)	11 (26.19)	
Dyspareunia			
Yes	29 (10)	11 (27.5)	**< 0.001**⁣^∗^
No	261 (90)	29 (72.5)	
Dysuria			
Yes	98 (33.79)	18 (18.37)	0.158⁣^∗^
No	192 (66.21)	22 (11.46)	
Vaginal itching			
Yes	123 (42.41)	30 (24.39)	**< 0.001**⁣^∗^
No	167 (57.59)	10 (5.99)	
Discharge presence			
Yes	215 (74.14)	40 (18.61)	**< 0.001** ^**#**^
No	75 (25.86)	0 (0)	
Discharge color			
Curdy-white	22 (7.59)	2 (9.09)	**< 0.001** ^**#**^
Greenish	47 (16.21)	21 (44.68)	
Normal	75 (25.86)	0 (0)	
Milky-white	121 (41.72)	8 (6.61)	
Mixed	25 (8.62)	9 (36)	
Foul-smelly			
Yes	94 (32.41)	34 (36.17)	**< 0.001**⁣^∗^
No	196 (67.59)	6 (3.06)	
Strawberry cervix			
Yes	7 (2.41)	7 (100)	**< 0.001**⁣^∗^
No	283 (97.59)	33 (11.66)	
Sex partner			
One	280 (96.55)	38 (13.57)	**0.016** ^**#**^
≥ 2	2 (0.69)	2 (100)	
Not active	8 (2.76)	0 (0)	
Contraceptive method			
Barrier	28 (9.65)	4 (14.29)	0.4308^**#**^
Hormonal	90 (31.04)	13 (14.44)	
IUDs	2 (0.69)	1 (50)	
Never use	151 (52.07)	22 (14.57)	
Permanent	11 (3.79)	0 (0)	
Not active	8 (2.76)	0 (0)	

*Note:* Bold emphasis indicates significance.

⁣^∗^Chi-square test.

^#^Fisher exact test while any cell has a count less than 5.

**Table 4:** Association between *T*. *vaginalis* infection and clinical symptoms using logistic regression analysis (*n* = 290).

**Reported symptoms and clinical presentation/characteristics**	**Univariate**	**Multivariate **⁣^∗^
	**cOR [95% CI]**	**aOR [95% CI]**
Body temperature		
< 98.6	Ref	Ref
> 98.6	**13.23 [5.94 to 29.48]**	**15.89 [5.07 to 49.76]**
Lower abdominal pain		
No	Ref	Ref
Yes	**0.43 [0.19 to 0.95]**	0.57 [0.16 to 1.97]
Dyspareunia		
No	Ref	Ref
Yes	**4.90 [2.10 to 11.36]**	2.07 [0.58 to 7.35]
Dysuria		
No	Ref	—
Yes	1.74 [0.88 to 3.42]	—
Vaginal itching		
No	Ref	Ref
Yes	**5.07 [2.37 to 10.83]**	2.36 [0.88 to 6.32]
Discharge color		
Others	Ref	Ref
Greenish	**9.52 [4.53 to 19.99]**	**7.49 [2.73 to 20.54]**
Foul-smelly		
No	Ref	Ref
Yes	**17.94[7.19 to 44.81]**	**10.18 [3.34 to 31.03]**

*Note:* Significant values are in bold. Strawberry cervix was not included in the model due to 100% positivity, which resulted in infinite odds ratios and unstable confidence intervals.

Abbreviations: 95% CI, 95% confidence interval; aOR, adjusted OR; BMI, body mass index; cOR, crude odds ratio; Ref, reference.

⁣^∗^Variables associated (*p* < 0.05) in the bivariate model were forcibly entered in the multivariable model, model fit *p* < 0.05. Significant, Nagelkerke *R* square: 0.583..

**Table 5:** Association between *T. vaginalis* infection, sociodemographic and behavioral characteristics using logistic regression analysis (*n* = 290).

**Sociodemographic and behavioral characteristics**	**Univariate**	**Multivariate **⁣^∗^
Variables	**cOR [95% CI]**	**aOR [95% CI]**
Age (years)	1.00 [0.95 to 1.05]	0.99 [0.89 to 1.10]
BMI (kg/m^2^)	1.03 [0.93 to 1.13]	0.85 [0.67 to 1.09]
Reported occupation		
Others	Ref	Ref
Worker	**2.59 [1.25 to 5.37]**	**9.36 [2.82 to 31.06]**
Reported social caste/class		
Others	Ref	Ref
Dalits	1.35 [0.55 to 3.29]	0.83 [0.12 to 5.95]
Reported educational status		
Some school/college	Ref	Ref
Cannot read write	0.54 [0.22 to 1.34]	0.60 [0.10 to 3.72]
Reported primary residency		
Urban	Ref	Ref
Rural	1.34 [0.61 to 2.96]	0.61 [0.17 to 2.17]
Reported income categories		
Medium/upper	Ref	Ref
Lower	0.84 [0.31 to 2.32]	0.38 [0.06 to 2.32]
Reported contraception methods		
Condoms/barrier	Ref	Ref
Other	0.82 [0.24 to 2.80]	0.90 [0.20 to 3.94]

*Note:* Significant values are in bold, multiple sexual partners were not included in the multivariable model due to 100% positivity, which resulted in infinite odds ratios and unstable confidence intervals.

Abbreviations: 95% CI, 95% confidence interval; aOR, adjusted OR; BMI, body mass index; cOR, crude odds ratio; Ref, reference.

⁣^∗^All variables in the bivariate model were forcibly entered in the multivariable model, model fit *p* < 0.05, significant, Nagelkerke *R* square: 0.280.

## Data Availability

The datasets generated and/or analyzed during the current study are not publicly available but are available from the corresponding author on reasonable request.
